# Association of cancer with the risk of developing hypertension

**DOI:** 10.1093/ehjqcco/qcad036

**Published:** 2023-06-15

**Authors:** Hajime Nagasawa, Hidehiro Kaneko, Yuta Suzuki, Akira Okada, Katsuhito Fujiu, Norifumi Takeda, Hiroyuki Morita, Akira Nishiyama, Yuichiro Yano, Koichi Node, Anthony J Viera, Robert M Carey, Suzanne Oparil, Hideo Yasunaga, Rhian M Touyz, Issei Komuro

**Affiliations:** The Department of Cardiovascular Medicine, The University of Tokyo, Tokyo, Japan; Department of Nephrology, Juntendo University Faculty of Medicine, Tokyo, Japan; The Department of Cardiovascular Medicine, The University of Tokyo, Tokyo, Japan; The Department of Advanced Cardiology, The University of Tokyo, Tokyo, Japan; The Department of Cardiovascular Medicine, The University of Tokyo, Tokyo, Japan; Center for Outcomes Research and Economic Evaluation for Health, National Institute of Public Health, Saitama, Japan; Department of Prevention of Diabetes and Lifestyle-Related Diseases, Graduate School of Medicine, The University of Tokyo, Tokyo, Japan; The Department of Cardiovascular Medicine, The University of Tokyo, Tokyo, Japan; The Department of Advanced Cardiology, The University of Tokyo, Tokyo, Japan; The Department of Cardiovascular Medicine, The University of Tokyo, Tokyo, Japan; The Department of Cardiovascular Medicine, The University of Tokyo, Tokyo, Japan; Department of Pharmacology, Faculty of Medicine, Kagawa University, Kagawa, Japan; Noncommunicable Disease (NCD) Epidemiology Research Center, Shiga University of Medical Science, Shiga, Japan; Department of Family Medicine and Community Health, Duke University, Durham, NC, USA; Department of Cardiovascular Medicine, Saga University, Saga, Japan; Department of Family Medicine and Community Health, Duke University, Durham, NC, USA; Department of Medicine, University of Virginia, Charlottesville, VA, USA; Division of Cardiovascular Disease, Department of Medicine, University of Alabama at Birmingham, Birmingham, AL, USA; The Department of Clinical Epidemiology and Health Economics, School of Public Health, The University of Tokyo, Tokyo, Japan; Research Institute, McGill University Health Centre, Montreal, Quebec, Canada; The Department of Cardiovascular Medicine, The University of Tokyo, Tokyo, Japan; International University of Health and Welfare, Tokyo, Japan

**Keywords:** Cancer, Hypertension, Epidemiology

## Abstract

**Background and aims:**

Although the importance of hypertension in patients with cancer is widely recognized, little is known about the risk of developing hypertension in patients with a history of cancer.

**Methods and results:**

This retrospective observational cohort study analysed data from the JMDC Claims Database between 2005 and 2022, including 78 162 patients with a history of cancer and 3692 654 individuals without cancer. The primary endpoint was the incidence of hypertension.

During a mean follow-up period of 1208 ± 966 days, 311 197 participants developed hypertension. The incidence of hypertension was 364.6 [95% confidence interval (CI) 357.0–372.2] per 10 000 person-years among those with a history of cancer, and 247.2 (95% CI 246.3–248.1) per 10 000 person-years in those without cancer. Individuals with a history of cancer had an elevated risk of developing hypertension, according to multivariable Cox regression analyses [hazard ratio (HR) 1.17, 95% CI 1.15–1.20]. Both cancer patients requiring active antineoplastic therapy (HR 2.01, 95% CI 1.85–2.20), and those who did not require active antineoplastic therapy (HR 1.14, 95% CI 1.12–1.17) had an increased risk of hypertension. A multitude of sensitivity analyses confirmed the robustness of the relationship between cancer and incident hypertension. Patients with certain types of cancer were found to have a higher risk of developing hypertension than those without cancer, with varying risks dependent on the type of cancer.

**Conclusion:**

Our analysis of a nationwide epidemiological database revealed that individuals with a history of cancer have a higher risk of developing hypertension, and this finding applies to both cancer patients who require active antineoplastic therapy and those who do not.

## Introduction

The clinical importance of hypertension in cancer patients has become widely recognized. This is related, in part, to the fact that the risk of both hypertension and cancer increases with increasing age, and the clear evidence that modern anti-cancer drugs cause hypertension.^1^ Accordingly, careful diagnosis and treatment of cancer therapy-related hypertension is essential in the management of patients receiving antineoplastic therapy. Hypertension is reported to occur in over one-half of cancer patients treated with vascular endothelial growth factor inhibitors due to decreased nitric oxide production and angiogenesis, oxidative stress, impaired natriuresis, endothelin-1-mediated vasoconstriction, and thrombotic microangiopathy.^2–5^ Alkylating and alkyl-like agents can induce hypertension via vascular endothelial injury and nephrotoxicity.^6,7^

Hypertension can be a paraneoplastic feature of patients with cancer. Cancer may facilitate the development of hypertension. Several case reports have shown that paraneoplastic hypertension in hepatocellular carcinoma can be driven by cancer cells producing excessive angiotensinogen or angiotensin I.^8,9^ More than 75% of patients with renal cell carcinoma also have hypertension.^10^ Endothelin-1 and other vasoactive peptides secreted from renal cell carcinoma cells are thought to mediate paraneoplastic hypertension.^11^ Further, recent epidemiological studies have reported that hypertension can be a risk factor for liver cancer and kidney cancer,^12–15^ but the causal relationship between hypertension and the incidence of these cancers remains unclear.

There are several potential molecular mechanisms by which some cancers could induce hypertension, but there has been so far scarce evidence on the relationship between cancer and incident hypertension,^16^ and epidemiological data have not been sufficient to determine whether cancer can be a risk factor for hypertension development in a real-world clinical setting. In particular, and in order to advance the field, it would be important to determine whether the risk of developing hypertension is also increased in cancer patients who are not receiving antineoplastic therapy such as vascular endothelial growth factor inhibitors and tyrosine kinase inhibitors, which mediate increases in blood pressure (BP). This study aimed to clarify the association between a history of cancer, in antineoplastic therapy-treated and untreated patients, and the incidence of hypertension using a nationwide epidemiological cohort.

## Methods

Access to the JMDC Claims Database is available from JMDC Inc.

### Study population

**Figure 1:**
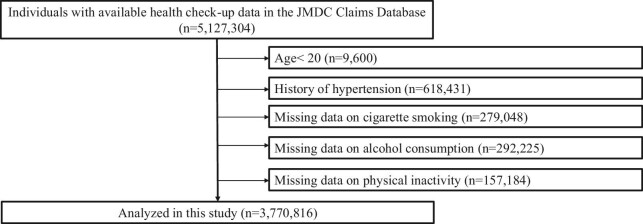
Flowchart. We identified 5127 304 individuals with available annual health check-up data from January 2005 through May 2022, including blood pressure, fasting plasma glucose, and lipid profile, >1 year after insurance enrolment (1-year look-back period). Individuals who met the following criteria were excluded: age < 20 years (*n* = 9600), prior history of hypertension (*n* = 618 431); missing data on cigarette smoking (*n* = 279 048); missing data on alcohol consumption (*n* = 292 225); and missing data on physical inactivity (*n* = 157 184). Finally, this cohort comprised 3770 816 participants.

In this retrospective cohort study, we reviewed data from the JMDC Claims Database (JMDC; Tokyo, Japan),^17–19^ which includes deidentified individual health insurance data (both outpatient and inpatient settings) for >60 insurers. The JMDC Claims Database covered individuals who were mainly employees working for relatively large companies in Japan under the coverage of the health insurance system for employees. The cumulative population is ∼16 million (as of May 2023). Annual health checkup data and administrative claims data for registered individuals were available in this dataset. Diagnostic coding based on the International Classification of Diseases, 10th Revision (ICD-10), was also recorded in this database. We identified 5127 304 individuals with available annual health check-up data from January 2005 to May 2022, including BP, fasting plasma glucose, and lipid profile, >1 year after insurance enrolment (1-year look-back period). Individuals who met the following criteria were excluded: age < 20 years (*n* = 9600), history of hypertension (defined as ICD-10 codes of I10–I15 at the initial health checkup) (*n* = 618 431), missing data on cigarette smoking (*n* = 279 048), missing data on alcohol consumption (*n* = 292 225), and missing data on physical inactivity (*n* = 157 184). The final cohort comprised 3770 816 participants (*Figure 1*).

### Ethics

This study was approved by the Ethics Committee of the University of Tokyo (2018-10862) and complied with the Declaration of Helsinki. Informed consent was not required because the JMDC Claims Database consisted of de-identified data.

### Definition of cancer history

Patients diagnosed with malignant neoplasms (ICD-10 codes: C00–D09) before the initial health check-up were defined as individuals with a history of cancer. We extracted information on the diagnosis of selected cancer types with a relatively higher incidence rate in the Japanese population (https://ganjoho.jp/reg_stat/statistics/stat/summary.html). Supplementary material lists the ICD-10 codes for each cancer diagnosis.

### Definition

Obesity was defined as a body mass index (BMI) ≥ 25 kg/m^2^. Diabetes mellitus was defined as fasting plasma glucose ≥ 126 mg/dL or the use of antidiabetic medications. Dyslipidaemia was defined as low-density lipoprotein cholesterol ≥ 140 mg/dL, high-density lipoprotein cholesterol < 40 mg/dL, triglyceride ≥ 150 mg/dL, or the use of antihyperlipidaemic medications. Smoking status (current or non-current/never) and alcohol consumption (daily or not) were extracted from a self-reported questionnaire survey. We also defined physical inactivity as not performing 30 min of exercise ≥ two times a week or not walking for >1 h per day.

### Outcomes

The clinical outcomes were collected between January 2005 and May 2022. Hypertension (ICD-10 code: I10–I15) was the primary endpoint. We followed study participants from the initial health check-up to the incident of hypertension, death, leaving insurance, or study end (May 2022).

### Statical procedures

Baseline characteristics are presented using medians with interquartile ranges (IQR) for continuous data and numbers (percentages) for categorical data. Differences between patients with a history of cancer and those without a history of cancer were compared using the Mann–Whitney *U* test and the chi-square test, as appropriate. The cumulative incidence of hypertension was described using the Kaplan–Meier method and compared using the log-rank test. The association between a history of cancer and the incidence of hypertension was examined using univariable and multivariable Cox proportional hazard regression models. Model 1 was a univariable model. Model 2 was a multivariable model adjusted for age, sex, BMI, systolic BP, diastolic BP, diabetes mellitus, dyslipidaemia, cigarette smoking, alcohol consumption, and physical inactivity. Study participants were divided into the following three groups: those who had no history of cancer, those who had a history of cancer but did not receive active antineoplastic therapy, and those who had a history of cancer and received active antineoplastic therapy. Active antineoplastic therapy was defined as the use of antineoplastic agents within 3 months of the initial health check-up based on the WHO-ATC code (WHO-ATC code: L01) because the Japanese system of universal health insurance allows for a 3-month maximum prescription term. Cox proportional hazard regression analysis was used to compare the risk of developing hypertension in three groups: individuals with no cancer history, those with cancer history but no active antineoplastic therapy, and those with cancer history and active antineoplastic therapy. We also conducted this analysis stratified by sex.

We estimated the HRs for incident hypertension among those who had the five cancer sites with the highest number of patients in our dataset, using the Cox proportional hazard regression model. For this analysis, we excluded patients with multiple cancers.

Seven sensitivity analyses were performed. First, multiple imputations using chained equations were performed to address missing data on covariates, including cigarette smoking, alcohol consumption, and physical inactivity. The results were aggregated over 20 imputed sets using Rubin's rules.^20,21^ Second, we used the Fine and Gray subdistribution hazard model to examine the association between cancer history and the incidence of hypertension because all-cause death could be considered a competing risk factor. Third, we excluded individuals with a diagnosis of hypertension based only on ICD-10 codes in the primary analysis. Therefore, we examined the association between cancer history and the incidence of hypertension after further excluding individuals with a systolic BP ≥ 140 mmHg or diastolic BP ≥ 90 mmHg at the initial health check-up in this sensitivity analysis. Fourth, we defined the outcome as the diagnosis of hypertension with a prescription of BP-lowering medications in the months before and after the diagnosis of hypertension. Fifth, we excluded study participants with <1 year of follow-up (i.e. 1-year induction period). Sixth, we added the estimated glomerular filtration rate as an adjustment variable in the multivariable model. Seventh, we conducted a subgroup analysis stratified by median age (≥43 years vs. < 43 years) (using a median age as a cut-off value), sex (men vs. women), and median BMI (≥22.1 kg/m^2^ vs. < 22.1 kg/m^2^) (using a median BMI as a cut-off value).

A two-sided *P* < 0.05 was considered statistically significant. All statistical analyses were performed using the Stata software (version 17; StataCorp LLC, College Station, TX, USA).

## Results

### Baseline characteristics

Of 3770 816 individuals, 78 162 (2.1%) had a history of cancer. *Table 1* presents the baseline characteristics of the study participants. At the initial health check-up, the median systolic and diastolic BP were 116 (106–126) and 71 (64–80) mmHg, respectively. The median age of the study participants was 43 years (IQR, 35–51 years), and 2118 101 (56.2%) were men. Compared with individuals without a history of cancer, individuals with a history of cancer were older and more likely to be women. The prevalence of diabetes mellitus and dyslipidaemia was higher in individuals with a history of cancer.

**Table 1:** Baseline characteristics

	Overall (*n* = 3770 816)	Cancer (–) (*n* = 3692 654)	Cancer (+) (*n* = 78 162)	*P*-value
Systolic blood pressure, mmHg	116 (106–126)	116 (106–126)	117 (106–128)	<0.001
Diastolic blood pressure, mmHg	71 (64–80)	71 (64–80)	72 (65–80)	<0.001
Age, years	43 (35–51)	43 (35–50)	52 (45–59)	<0.001
Men, *n* (%)	2118 101 (56.2)	2088 994 (56.6)	29 107 (37.2)	<0.001
Body mass index, kg/m^2^	22.1 (20–24.6)	22.1 (20.1–24.6)	21.7 (19.7–24.1)	<0.001
Overweight, *n* (%)	832 289 (22.1)	817 815 (22.1)	14 474 (18.5)	<0.001
Diabetes mellitus, *n* (%)	84 052 (2.2)	81 512 (2.2)	2540 (3.2)	<0.001
Dyslipidaemia, *n* (%)	1382 136 (36.7)	1349 961 (36.6)	32 175 (41.2)	<0.001
Cigarette smoking, *n* (%)	951 824 (25.2)	941 620 (25.5)	10 204 (13.1)	<0.001
Alcohol consumption, *n* (%)	787 597 (20.9)	772 277 (20.9)	15 320 (19.6)	<0.001
Physical inactivity, *n* (%)	1936 727 (51.4)	1896 106 (51.3)	40 621 (52.0)	<0.001
Fasting plasma glucose, mg/dL	91 (85–97)	91 (85–97)	92 (87–99)	<0.001
Low-density lipoprotein cholesterol, mg/dL	118 (98–140)	118 (98–140)	120 (99–142)	<0.001
High-density lipoprotein cholesterol, mg/dL	62 (52–74)	62 (52–74)	67 (56–80)	<0.001
Triglycerides, mg/dL	77 (55–116)	77 (55–116)	79 (57–114)	<0.001

Values are shown as *n* (%) or median (interquartile range). *P-*values were calculated using a chi-square test for categorical variables and a Mann–Whitney *U* test for continuous variables.

### Cancer history and incidence of hypertension

**Figure 2:**
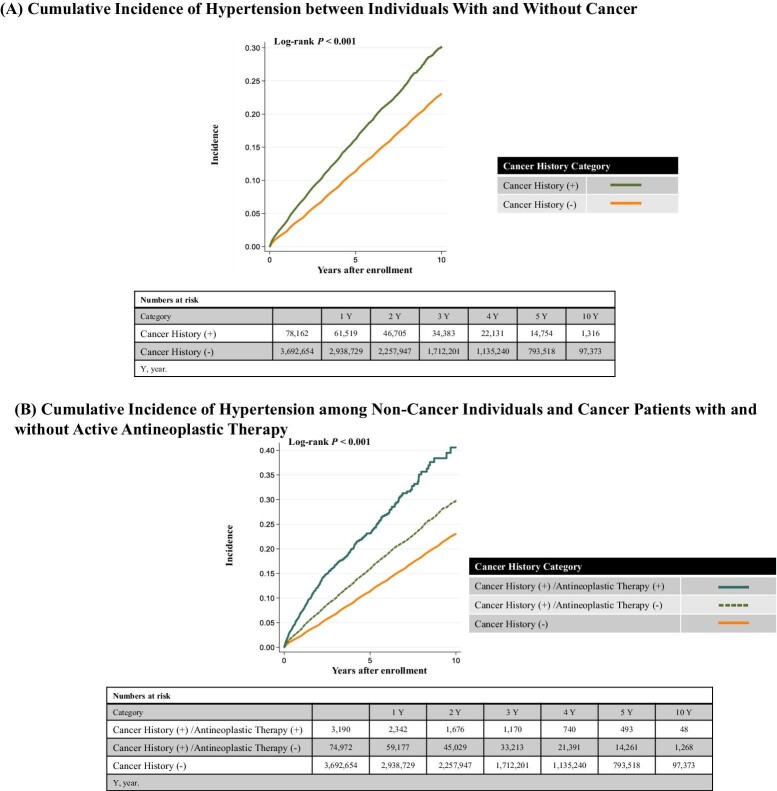
Kaplan–Meier curves. The cumulative incidence of hypertension was examined using the Kaplan–Meier method. The difference in incidence of hypertension according to the presence of cancer history was compared using the log-rank test (*A*). The difference in incidence of hypertension according to the presence of cancer history and active antineoplastic therapy was compared using the log-rank test (*B*).

The study participants were followed up for a mean of 1208 ± 966 days. During the follow-up period, 311 197 participants developed hypertension. The loss to follow-up, which is defined as leaving insurance before May 2022, was seen in 974 752 individuals. Compared with individuals without a history of cancer, the cumulative incidence of hypertension was higher in individuals with a history of cancer (log-rank test, *P* < 0.001) (*Figure 2A*). The incidence of hypertension was 364.6 (357.0–372.2) per 10 000 person-years in individuals with a history of cancer, and 247.2 (246.3–248.1) per 10 000 person-years in those without a history of cancer. In the univariate model (model 1), a history of cancer was associated with a higher risk of hypertension. In the multivariable analysis (model 2), individuals with a history of cancer had a higher risk of hypertension compared with individuals without a history of cancer [hazard ratio (HR) 1.17, 95% confidence interval (95% CI) 1.15–1.20] (*Table 2*). Compared with individuals without a history of cancer, the cumulative incidence of hypertension was higher in cancer patients without active antineoplastic therapy and further increased in cancer patients receiving active antineoplastic therapy (log-rank test, *P* < 0.001) (*Figure 2B*). Compared with individuals without a history of cancer, those who had a history of cancer and received active antineoplastic therapy showed the highest risk of hypertension, followed by those who had a history of cancer but did not receive active antineoplastic therapy. These results remained regardless of sex (*Table 3*).

**Table 2:** The frequency of events, corresponding incidence rates, and hazard ratios for hypertension events among participants by cancer

	Cancer (–)	Cancer (+)
Number	3692 654	78 162
Number of hypertension events	302 344	8853
Incidence (per 10 000 person-years)	247.2 (246.3–248.1)	364.6 (357.0–372.2)
Model 1 (95% confidence interval)	1 (reference)	1.48 (1.45–1.51)
Model 2 (95% confidence interval)	1 (reference)	1.17 (1.15–1.20)

Model 1 = unadjusted. Model 2 = adjusted for age, sex, body mass index, systolic blood pressure, diastolic blood pressure, diabetes mellitus, dyslipidaemia, cigarette smoking, alcohol consumption, and physical inactivity. Participants were categorized into two groups according to the absence or presence of cancer.

**Table 3:** The frequency of events, corresponding incidence rates, and hazard ratios for hypertension events among participants by cancer and active antineoplastic therapy

	Cancer (–)	Cancer (+) Antineoplastic aherapy (–)	Cancer (+) Antineoplastic therapy (+)
Number	3692 654	74 972	3190
Number of hypertension events	302 344	8334	519
Incidence (per 10 000 person-years)	247.2 (246.3–248.1)	356.1 (348.5–363.8)	589.0 (540.4–642.0)
Model 1 (95% confidence interval)	1 (reference)	1.45 (1.42–1.48)	2.39 (2.20–2.61)
Model 2 (95% confidence interval)	1 (reference)	1.14 (1.12–1.17)	2.01 (1.85–2.20)
Men			
Number	2088 994	27 534	1573
Number of hypertension events	207 792	4321	315
Incidence (per 10 000 person-years)	288.2 (286.9–289.4)	489.0 (474.6–503.8)	725.4 (649.5–810.1)
Model 1 (95% confidence interval)	1 (reference)	1.70 (1.65–1.76)	2.54 (2.27–2.83)
Model 2 (95% confidence interval)	1 (reference)	1.18 (1.14–1.22)	2.13 (1.91–2.38)
Women			
Number	1603 660	47 438	1617
Number of hypertension events	94 552	4013	204
Incidence (per 10 000 person-years)	188.4 (187.2–189.6)	275.5 (267.1–284.2)	456.5 (397.9–523.6)
Model 1 (95% confidence interval)	1 (reference)	1.47 (1.42–1.51)	2.42 (2.11–2.78)
Model 2 (95% confidence interval)	1 (reference)	1.11 (1.08–1.15)	1.87 (1.63–2.15)

Model 1 = unadjusted. Model 2 = adjusted for age, sex, body mass index, systolic blood pressure, diastolic blood pressure, diabetes mellitus, dyslipidaemia, cigarette smoking, alcohol consumption, and physical inactivity. Participants were categorized into three groups: cancer history absent, cancer history present without active antineoplastic therapy, and cancer history present with active antineoplastic therapy. Subgroup analyses stratified by sex excluded sex from covariates.

The five cancer sites with the greatest number of affected male individuals were colorectal cancer (*n* = 5744), stomach cancer (*n* = 3989), prostate cancer (*n* = 2845), lung cancer (*n* = 1851), and non-Hodgkin lymphoma (*n* = 1801). The five cancer sites with the greatest number of affected women were breast cancer (*n* = 19 601), thyroid cancer (*n* = 4065), colorectal cancer (*n* = 3706), cervix uteri cancer (*n* = 3592), and corpus uteri cancer (*n* = 2516). Compared with non-cancer participants, the HR (95% CI) for hypertension in men with colorectal cancer, stomach cancer, prostate cancer, lung cancer, and non-Hodgkin lymphoma were 1.14 (1.07–1.22), 1.15 (1.06–1.24), 1.13 (1.03–1.23), 1.24 (1.11–1.39), and 1.14 (1.00–1.30), respectively. Compared with non-cancer participants, the HR (95% CI) for hypertension in women with breast cancer, thyroid cancer, colorectal cancer, cervix uteri cancer, and corpus uteri cancer were 1.05 (1.00–1.11), 1.22 (1.10–1.35), 1.11 (1.00–1.23), 1.16(1.02–1.30), and 1.19 (1.05–1.35), respectively (Supplementary material online, *Figure S1*).

### Sensitivity analyses

Seven sensitivity analyses were conducted. First, we imputed the missing data on covariates using multiple imputations and studied 4499 273 individuals. Individuals with a history of cancer had a higher risk of hypertension compared with those without a history of cancer (Supplementary material online, *Table S1*). Second, we used the Fine and Gray subdistribution hazard models. The significant association between a history of cancer and the incidence of hypertension was unchanged (Supplementary material online, *Table S2*). Third, our main findings remained unchanged even after excluding individuals with systolic BP ≥ 140 mmHg or diastolic BP ≥ 90 mmHg at the initial health checkup (*n* = 373 644) (Supplementary material online, *Table S3*). Fourth, we redefined the outcome as the diagnosis of hypertension with a prescription of BP-lowering medications in the months before and after the diagnosis of hypertension. Individuals with a history of cancer also had a higher risk of hypertension compared with those without a history of cancer (Supplementary material online, *Table S4*). Fifth, the results of the main analysis were unchanged, even if we set a 1-year induction period (Supplementary material online, *Table S5*). Sixth, we analysed 1968 502 patients with available data on estimated glomerular filtration rate, and our primary findings were consistent after adjustment for estimated glomerular filtration rate (Supplementary material online, *Table S6*). Seventh, a history of cancer was associated with a greater risk of hypertension across all subgroups (stratified by age, sex, and BMI) (Supplementary material online, *Figure S2*).

## Discussion

In this nationwide analysis of a health claims database including adults who underwent annual health checkups, the cumulative incidence of hypertension was higher in individuals with a history of cancer compared with those without. Individuals with and without active antineoplastic therapy for cancer have a higher risk of developing hypertension. We confirmed the robustness of our primary findings using a multitude of sensitivity analyses. To the best of our knowledge, this is the first study to uncover the relationship between cancer and a greater risk of hypertension using a large-scale epidemiological dataset.

Our study has several novel clinical implications. First, our study showed that a history of cancer is associated with a higher risk of developing hypertension. Consistent with previous studies, cancer patients receiving active antineoplastic therapy had an increased risk of hypertension; however, it is noteworthy that cancer patients who did not receive active antineoplastic therapy also had a greater risk of developing hypertension compared with non-cancer participants. This result remained unchanged after adjusting for covariates. As reported in preceding investigations, the use of anticancer drugs greatly increased the risk of developing hypertension, but our data suggest that cancer itself may also increase the risk of developing hypertension. Second, the Kaplan–Meier curve showed that the difference in the risk of developing hypertension between patients with a history of cancer and those without a history of cancer continued to widen without attenuation over time. This finding indicates that long-term BP monitoring is important for cancer survivors and that short-term BP monitoring (e.g. during active antineoplastic therapy) is not sufficient. Prevention of cardiovascular disease in cancer survivors is essential to improve their prognosis. Given that hypertension is the leading cause of cardiovascular disease and that hypertension is strongly associated with an increased risk of cardiovascular disease even in cancer survivors,^22^ the findings of the current study suggest that incident hypertension in cancer survivors may exist in the background of the development of cardiovascular disease, highlighting a potential cancer-hypertension-cardiovascular disease axis. Thus, BP monitoring must remain a key preventive care strategy for cancer survivors. Third, we analysed the individual cancer types and the subsequent risk of hypertension. We found that patients with individual cancer types were at a higher risk of developing hypertension compared with people without a history of cancer, but the risk was not uniform. For example, in men, patients with lung cancer appeared to have a higher risk of developing hypertension compared with those with other cancer types, whereas in women, patients with breast cancer had a relatively lower risk of hypertension compared with women with other cancer types. While the causes of hypertension are diverse in the general population, triggers for hypertension are even more diverse in patients with cancer, including background clinical factors, cancer site, cancer stage, antineoplastic therapy, concomitant medications, pain, and anxiety due to cancer. Further epidemiological data should be accumulated to enable a refined assessment of the risk of developing hypertension in patients with cancer and to establish individualized BP management for cancer survivors.

### Limitations

This study has some limitations. Due to the retrospective observational nature of this study, we could not conclude a causal relationship between cancer and hypertension and should acknowledge various potential biases. In particular, the potential presence of detection bias should be recognized because cancer patients visit medical providers more frequently than patients without cancer, which could lead to more opportunities to be diagnosed with hypertension. Although we conducted multivariable Cox regression analyses, the baseline clinical characteristics of study participants were different between participants with and without cancer, which could have affected our primary findings. We identified hypertension and cancer using ICD-10 codes registered in the JMDC Claims Database. Generally, the recorded diagnoses of administrative databases, including the JMDC Claims Database, are considered to be less validated. However, a previous study reported that the accuracy of the recorded diagnoses (including cancer and hypertension) in administrative databases in Japan is high.^23–26^ The JMDC Claims Database does not include people aged >75 years, and therefore, whether our findings could be applicable to older people is unclear. Because the cancer distribution among the Japanese population differs from that of most of the rest of the world, we cannot generalize our results. Detailed information (e.g. stage of cancer) was unavailable in this dataset. Finally, although our study suggests a potential association between cancer and a higher risk of hypertension, the underlying mechanisms of our findings should be uncovered by further investigation. Several studies have reported that hypertension could also increase the risk of developing specific cancers.^12–15^ Therefore, the mutual causal and pathophysiological association between cancer and hypertension is of great clinical interest.

## Conclusions

Using a nationwide population-based database, this analysis showed that patients with a history of cancer have a greater risk of developing hypertension. Further, not only cancer patients with active antineoplastic therapy but also those without are at a higher risk for hypertension development, suggesting that cancer itself could increase the risk of developing hypertension. Given the clinical significance of hypertension in cancer survivors, we need to identify the optimal BP management strategy for cancer survivors to improve the clinical outcomes of patients living with cancer. We believe that the results of this study confirm the importance of the novel scientific field of ‘onco-hypertension’.

## Supplementary Material

qcad036_Supplemental_File
