# Supplementary material for: Association of cancer with the risk of developing hypertension
Source: Eur Heart J Qual Care Clin Outcomes. 2023 Jun 15;10(3):228–34. doi: 10.1093/ehjqcco/qcad036 (PMC11112520; doi:10.1093/ehjqcco/qcad036)
Supplement: qcad036_Supplemental_File [file qcad036_supplemental_file.pptx]

## Slide 1
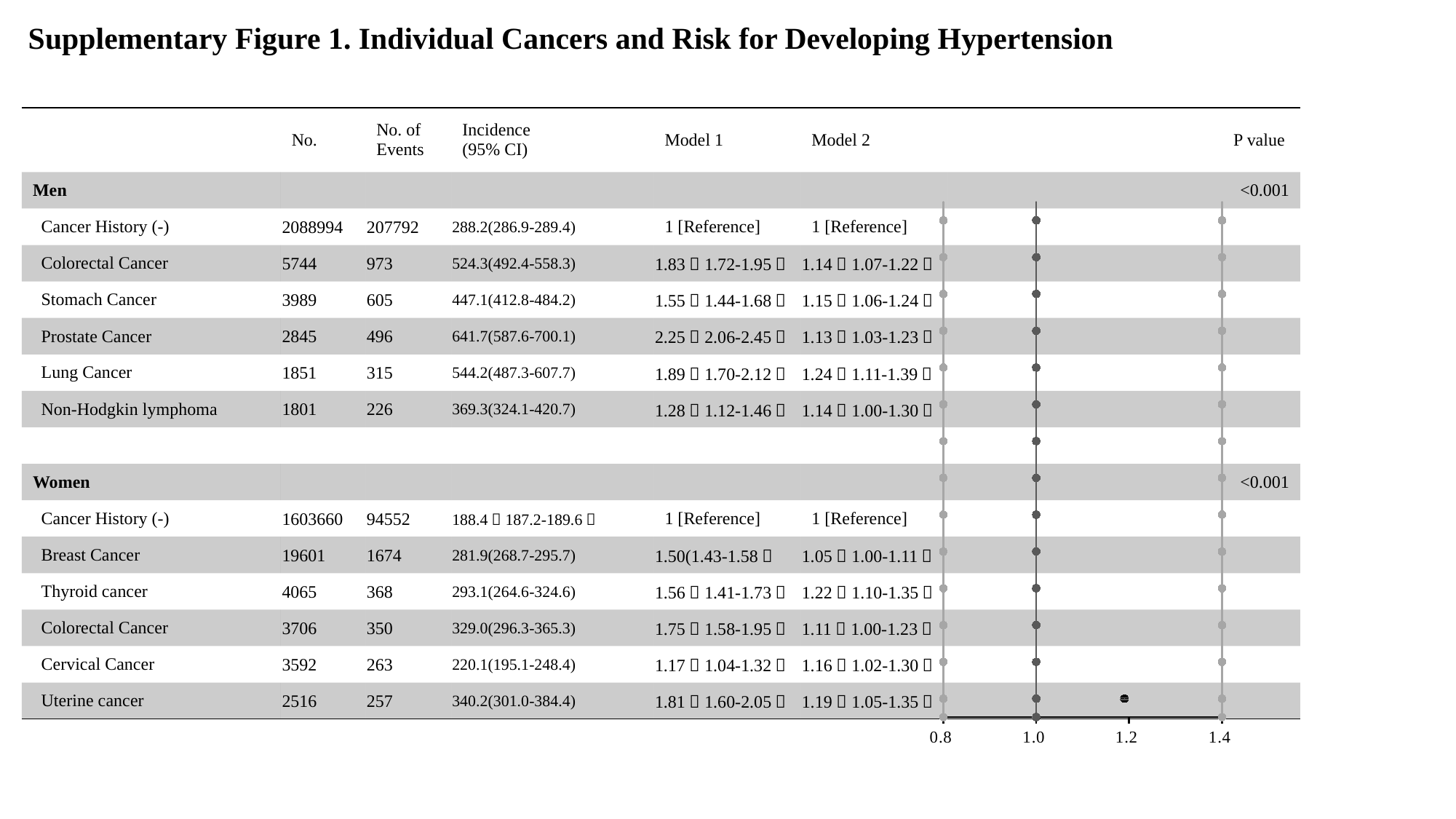

Supplementary Figure 1. Individual Cancers and Risk for Developing Hypertension
| | No. | No. of Events | Incidence (95% CI) | Model 1 | Model 2 | P value |
| --- | --- | --- | --- | --- | --- | --- |
| Men | | | | | | <0.001 |
| Cancer History (-) | 2088994 | 207792 | 288.2(286.9-289.4) | 1 [Reference] | 1 [Reference] | |
| Colorectal Cancer | 5744 | 973 | 524.3(492.4-558.3) | 1.83（1.72-1.95） | 1.14（1.07-1.22） | |
| Stomach Cancer | 3989 | 605 | 447.1(412.8-484.2) | 1.55（1.44-1.68） | 1.15（1.06-1.24） | |
| Prostate Cancer | 2845 | 496 | 641.7(587.6-700.1) | 2.25（2.06-2.45） | 1.13（1.03-1.23） | |
| Lung Cancer | 1851 | 315 | 544.2(487.3-607.7) | 1.89（1.70-2.12） | 1.24（1.11-1.39） | |
| Non-Hodgkin lymphoma | 1801 | 226 | 369.3(324.1-420.7) | 1.28（1.12-1.46） | 1.14（1.00-1.30） | |
| | | | | | | |
| Women | | | | | | <0.001 |
| Cancer History (-) | 1603660 | 94552 | 188.4（187.2-189.6） | 1 [Reference] | 1 [Reference] | |
| Breast Cancer | 19601 | 1674 | 281.9(268.7-295.7) | 1.50(1.43-1.58） | 1.05（1.00-1.11） | |
| Thyroid cancer | 4065 | 368 | 293.1(264.6-324.6) | 1.56（1.41-1.73） | 1.22（1.10-1.35） | |
| Colorectal Cancer | 3706 | 350 | 329.0(296.3-365.3) | 1.75（1.58-1.95） | 1.11（1.00-1.23） | |
| Cervical Cancer | 3592 | 263 | 220.1(195.1-248.4) | 1.17（1.04-1.32） | 1.16（1.02-1.30） | |
| Uterine cancer | 2516 | 257 | 340.2(301.0-384.4) | 1.81（1.60-2.05） | 1.19（1.05-1.35） | |
### Chart
| Category | 34 | | | | |
|---|---|---|---|---|---|

## Slide 2
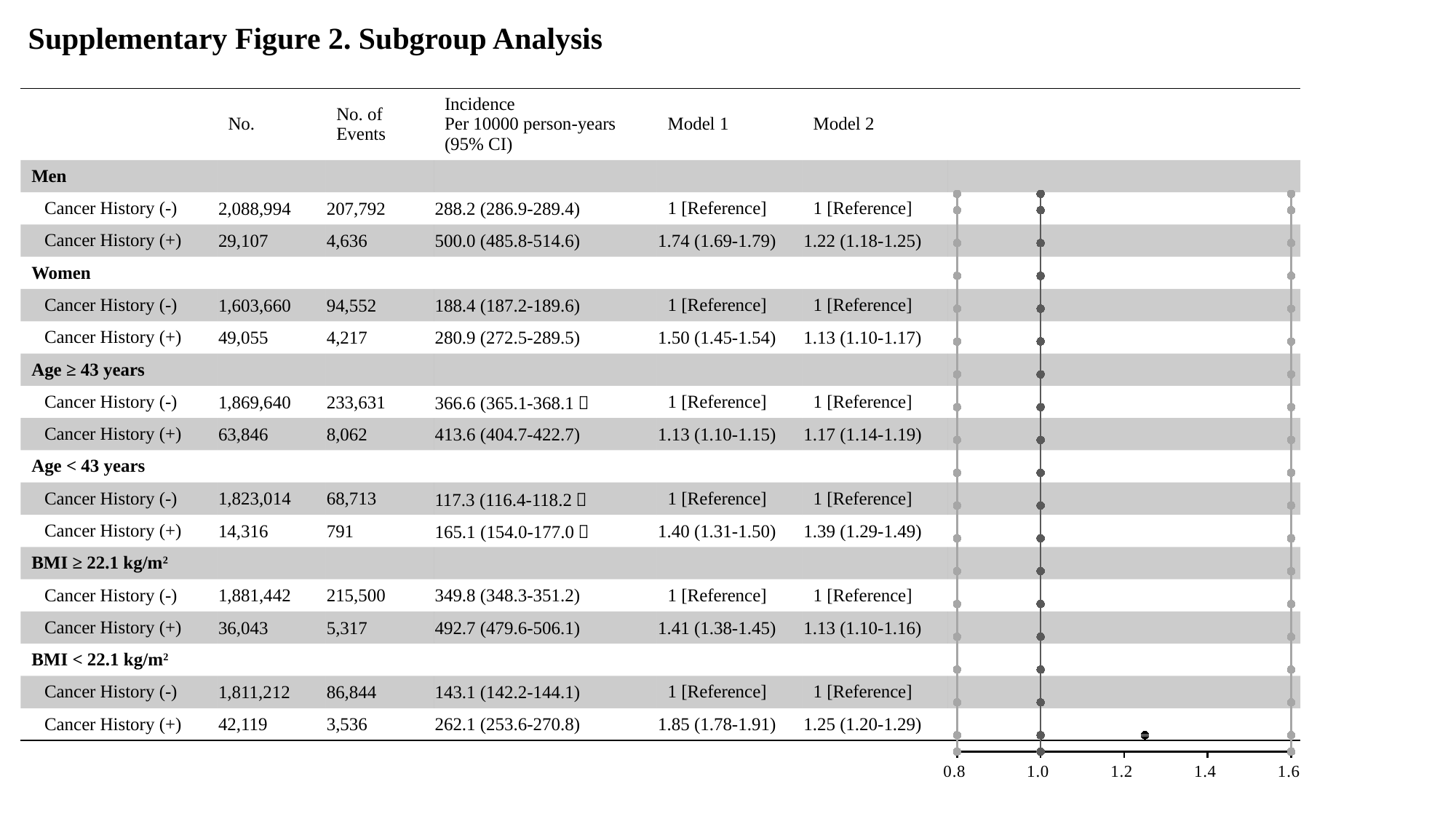

Supplementary Figure 2. Subgroup Analysis
| | No. | No. of Events | Incidence Per 10000 person-years (95% CI) | Model 1 | Model 2 | |
| --- | --- | --- | --- | --- | --- | --- |
| Men | | | | | | |
| Cancer History (-) | 2,088,994 | 207,792 | 288.2 (286.9-289.4) | 1 [Reference] | 1 [Reference] | |
| Cancer History (+) | 29,107 | 4,636 | 500.0 (485.8-514.6) | 1.74 (1.69-1.79) | 1.22 (1.18-1.25) | |
| Women | | | | | | |
| Cancer History (-) | 1,603,660 | 94,552 | 188.4 (187.2-189.6) | 1 [Reference] | 1 [Reference] | |
| Cancer History (+) | 49,055 | 4,217 | 280.9 (272.5-289.5) | 1.50 (1.45-1.54) | 1.13 (1.10-1.17) | |
| Age ≥ 43 years | | | | | | |
| Cancer History (-) | 1,869,640 | 233,631 | 366.6 (365.1-368.1） | 1 [Reference] | 1 [Reference] | |
| Cancer History (+) | 63,846 | 8,062 | 413.6 (404.7-422.7) | 1.13 (1.10-1.15) | 1.17 (1.14-1.19) | |
| Age < 43 years | | | | | | |
| Cancer History (-) | 1,823,014 | 68,713 | 117.3 (116.4-118.2） | 1 [Reference] | 1 [Reference] | |
| Cancer History (+) | 14,316 | 791 | 165.1 (154.0-177.0） | 1.40 (1.31-1.50) | 1.39 (1.29-1.49) | |
| BMI ≥ 22.1 kg/m2 | | | | | | |
| Cancer History (-) | 1,881,442 | 215,500 | 349.8 (348.3-351.2) | 1 [Reference] | 1 [Reference] | |
| Cancer History (+) | 36,043 | 5,317 | 492.7 (479.6-506.1) | 1.41 (1.38-1.45) | 1.13 (1.10-1.16) | |
| BMI < 22.1 kg/m2 | | | | | | |
| Cancer History (-) | 1,811,212 | 86,844 | 143.1 (142.2-144.1) | 1 [Reference] | 1 [Reference] | |
| Cancer History (+) | 42,119 | 3,536 | 262.1 (253.6-270.8) | 1.85 (1.78-1.91) | 1.25 (1.20-1.29) | |
### Chart
| Category | 34 | | | | |
|---|---|---|---|---|---|
